# Marketing Up the Wrong Tree? Organisational Perspectives on Attracting and/or Retaining Older Adults in Sport

**DOI:** 10.3389/fspor.2021.772361

**Published:** 2021-11-26

**Authors:** Claire Jenkin, Jannique G. Z. van Uffelen, Grant O'Sullivan, Jack Harvey, Rochelle M. Eime, Hans Westerbeek

**Affiliations:** ^1^Institute of Sport, School of Life and Medical Sciences, University of Hertfordshire, Hatfield, United Kingdom; ^2^Institute for Health and Sport, Victoria University, Melbourne, VIC, Australia; ^3^Physical Activity, Sports and Health Research Group, Department of Movement Sciences, KU Leuven - University of Leuven, Leuven, Belgium; ^4^School of Science, Psychology and Sport, Federation University, Ballarat, VIC, Australia

**Keywords:** older adults, sports participation, age-active, sport policy, age-friendly

## Abstract

Community sport is seen as a suitable setting for physical activity for different population groups. Older adults (aged 50+ years) are a rapidly growing population group. Physical activity is critical for healthy ageing, however sport participation rates for older adults are very low. The aim of this study was to investigate how sporting organisations perceive sport for older adults. This cross-sectional study surveyed 171 representatives from Australian National and State Sporting Organisations. Descriptive statistics were used to summarise the results and the three sporting organisation categories' (high, medium, and low participation) responses were compared using non-parametric statistics. Contextualised in the perspective of organisational change, a framework for marketing to the ageing consumer was used to interpret the results. Older adults are not a high priority group for most sporting organisations, however the benefits of engaging older adults were recognised, particularly in context of increasing participation numbers. A lack of age-appropriate programmes was perceived to be a major barrier of engaging older adults. This lack of programmes stems from older adults being deemed as a less attractive segment than other age groups for sporting organisations. Modifications that sports felt they could make to attract and/or retain older adults included specific marketing and age appropriate opportunities. There was widespread consensus across sporting organisations, suggesting that perceptions of older adult sport participation were comparable across the sector, such as increasing participation numbers and engaging their older fan base. In the context of attracting, and retaining, older adults in sport clubs, it was concluded that most sporting organisations are not (yet) ready to build “age friendly” sporting environments. There is very limited literature on the organisational perspective of older adults and sport, meaning this study is unique in the field. Although sport policy encourages organisations to grow their participation, most organisations do not actively and strategically engage older adults. This research provides an understanding of why this untapped market is not a priority target and provides comprehensive insights for policy makers to better engage with this population group.

## Introduction

From national to local community contexts, sporting organisations are expected to deliver sporting opportunities for a variety of different population groups across the lifespan. A major policy focus in many countries, including Australia, has been to increase overall participation numbers (Sport Australia, 2018). In line with these objectives, there are many different layers of sport governanace and administration for community sport. At a federal level in Australia, Sport Australia (SA) is the national government agency responsible for setting the national sport policy agenda for both elite and community level sport. National Sporting Organisations (NSOs) for individual sports are affiliated with SA, and they operate in alignment to SA policies, funding and strategic foci. Most NSOs have state counterparts in the eight States/Territories of Australia, and community sport clubs are registered with these governing bodies. In Australia, most organised sport participants at the community level are engaged through community sport clubs (Eime et al., 2020).

There is an increasing body of evidence that the far majority of people who participate in sport at community sport clubs are children and youth (Eime et al., 2016b; Westerbeek et al., 2019; Westerbeek, 2021). A study of 520,102 sport participants within the Australian state of Victoria, reported that nearly 80% of sport participants were under 30 years old, and nearly a third (28%) of all participants were aged 10–14 years (Eime et al., 2016b). The study also reported that in adulthood, participation was shown to decline, with <10% of sport club participants (age range 4–100 years) aged 50 years or over (hereafter categorised as older adults). This trend of decreasing participation in organised sport with increasing age is consistent internationally, for example in Spain (Palacios-Ceña et al., 2012).

Internationally, the sporting focus on young people for both elite and community sport, is largely influenced by national sport policy (Westerbeek, 2021). Community sport has also become an increasingly important public policy area across the globe over the last 20 years (Green and Collins, 2008; Westerbeek, 2021), especially for improving health (Khan et al., 2012; World Health Organisation, 2021). The most recent national sport policy in Australia is the Sport 2030 policy (Sport Australia, 2018). For community sport, this policy prioritises participation to improve Australians' health. Whilst it promotes community sport participation for Australians of all ages and is less youth focused than previous strategies, there is still a distinct focus on early childhood and school-aged children. This focus on young people is not limited to the across-sport perspective of SA, with numerous Australian NSOs similarly strategically focusing on young people.

Although sport is largely targeted at children and is popular in this age group (Casey et al., 2012; Eime et al., 2016a; Westerbeek, 2021), sporting organisations are under increasing pressure from sport policymakers to increase their overall participation numbers (Westerbeek, 2021). Sporting organisations are in a position to start developing target strategies that may attract other population groups, such as older adults, to enable business growth. Older adults are an attractive market for a number of reasons. Firstly, as previously mentioned, very few older adults currently play sport (Eime et al., 2016b), meaning they are a largely untapped market for most sports. Secondly, the size of the market segment is increasing as older adults are a rapidly growing population group. The proportion of older adults in Australia is currently 33% (Australian Bureau of Statistics, 2020), and is predicted to increase to ~39% by the year 2060 (Australian Bureau of Statistics, 2015). Finally, an ageing population is likely to lead to an increased burden on health and social care resources (Prince et al., 2015). Government departments and organisations responsible for health and/or sport, are showing a rising interest in facilitating older adults to become more physically active. The overriding objective is to encourage healthy ageing to reduce healthcare costs in the process (see for example Tam, 2011). Therefore, it seems that to leverage the health benefits of physical activity and promote sport as a leisure-time physical activity option for older adults, may be an untapped opportunity for community sporting organisations.

However, previous literature has identified potential barriers or deterrents to enable this untapped market to participate more in community sport. One relevant deterrent identified was non-inclusive marketing (Jenkin et al., 2021), where it was documented that most adverts were targeted at younger people, meaning older adults did not always feel welcome in sport. As outlined by Stroud and Walker (2013), it is essential to realise that marketing actions directed at older adults cannot be limited to framing these in context of either chronological age, lifestage or psychological age. Rather, they argue that “physiological ageing”—the way in which the mind, the body and the senses change—would be a valid approach in that it can be broken up in sensory, cognitive and physical components.

From the perspective of older adults in that regard, to use existing sport clubs (Brown and van Uffelen, 2019), and for clubs to diversify and grow their participation base (Sport Australia, 2017), the clubs' sporting and volunteering opportunities would require adaptation and extension to service the specific sensory, cognitive and physical needs of older adults. As sport policy focus has been on attracting and catering for young people in sport clubs, the current marketing, recruitment, retention, development, delivery and governance structures and strategies of sport, are ill equipped to service and retain large numbers of older adults.

Although some sporting organisations have started to sporadically target the older adult market through a range of different initiatives, for example, through modified sport specifically for older adults, this age group does not form a core participation market for the vast majority of sporting organisations. To develop sustainable processes towards a more targeted approach to increase sport participation by older adults, it is likely that a significant organisational change process has to occur. This process is likely to be initiated and then driven by policy changes formulated by national organisations such as Sport Australia. The development of modified products suggests that some organisations have considered their own readiness to change, but other sporting organisations will need to ensure their organisational processes, structures and resources are re-aligned to achieve long term success regarding older adult sport participation (Kotter, 1995; Casey et al., 2009). To develop and implement modified products to successfully engage this untapped market in the long term, would only be one part of a larger transformation of organisations. It would also require changes to their organisational practises, in response to having to broaden their market focus from youth only, to one that considered the whole lifespan. Understanding the concepts that underpin organisational change is vital for any organisation wanting to change their strategic outlook (Todnem By, 2005), and organisational change theories can assist with this. Beyond organisational change, the key driver to more successfully engaging with a new and rapidly growing target segment (older adults) is to introduce and operationalise the concept of creating “age-friendly” environments, and “age-friendly customer journeys” (Stroud and Walker, 2013).

Existing sport management research on organisational change theory has mainly focused on planned or paradoxical changes (Kikulis et al., 1992; Amis et al., 2004; Thibault and Babiak, 2005). Planned change refers to a change that an organisation creates and implements to gain a competitive advantage over their rivals (Slack and Parent, 2006), whilst an organisation that must change to remain competitive engages in paradoxical change (Slack and Parent, 2006). Planned change is usually influenced by both external and internal forces for change. For example, when reviewing organisational changes in Canada's sport system, Thibault and Babiak (2005) argued that external pressures from the national government and the media, coupled with the internal desire to change, resulted in a successful shift to an athlete centred operational approach.

Paradoxical change can be seen where external parties have forced sporting organisations to change their organisational policies (Slack and Hinings, 1992). This can include a change in government policy (Kikulis et al., 1992) and/or changes in organisational priority (Skinner et al., 1999). A most extreme case of paradoxical change in sport is upon us with the COVID-19 pandemic, forcing sporting organisations to re-consider the whole of their business models. Ironically, in context of this paper, targeting untapped markets will be one of the solutions for sport to recover following the impact of the pandemic. Thus, for either a planned or paradoxical organisational change to be successful, an organisation first needs to ensure they are prepared for that change and then they must have the relevant processes in place to successfully implement this change (Oakland and Tanner, 2007; Casey et al., 2012). For both types of change, external forces have been the primary, or at least a strong contributing driver, particularly from national governments. Therefore, the concepts of planned and paradoxical change through the influence of external forces, is used to contextualise this study. As most sporting organisations would need to undertake either a planned or paradoxical change to increase older adult participation, organisational change theory will be used to firstly understand how NSOs and State Sporting Organisations (SSOs) perceive sport for older adults, before proposing a simple marketing model for older adults to explore how these organisations can engage with this growing and relatively untapped market.

To date, most of the, albeit limited, research on older adults and the reasons their engagement in and with sport, has largely focused on participation from an older adult perspective. For example, there has been research undertaken at an elite, competitive level (Dionigi, 2006; Pike, 2012), for general community sport (Berg et al., 2015; Jenkin et al., 2018, 2021) or for specific community sports, such as softball (Naar et al., 2017), curling (Stone et al., 2018) and pickleball (Buzzelli and Draper, 2020). These studies can provide sporting organisations and policy makers with useful insights into older adult sport participation from an older adult perspective. However, there is little research on older adult sport participation from the organisational perspective. To that end we propose a simple model (adapted from Stroud and Walker, 2013) with three strategic options on how to respond to the ageing consumer. We use this model (presented in Table 1) to interpret our results. Stroud and Walker argue that although organisations have the option not to consider the special needs of older consumers, few of them will not have to increasingly interact with the growing cohort of senior citizens. Beyond this direct contact are their children and grandchildren who have a stake in their welfare and well-being. From a marketing point of view this means that organisations will have to allocate resources and invest in marketing support, in order to communicate and engage with the older consumer. In order to classify sporting organisations in the discussion part of the paper, we have taken the work of Stroud and Walker (2013) to distinguish between those sporting organisations that just let things happen – those that are passive in their approach to engaging older adults as customers, and those that are more pro-active creating age-friendly or even bespoke products and services for older adults.

**Table 1:** Strategic organisational options of how to respond to older adult sport consumers.

	**Wait for others to take action and replicate (passive)**	**Be relevant to adults aged 50+ and work towards age-friendly sporting environments**	**Be age-active and develop bespoke (physiological ageing informed) marketing campaigns**
Strategic action that can be taken	*Sit back and remain focused on young target segments*	*Create age-friendly sporting experiences*	*Develop focused and bespoke new products for adults aged 50+*
Likely outcome from action taken	*Missing the mark and missing the market opportunity*	*Future proofing the sport business*	*Actively exploiting the growth opportunity*

Informed by organisational change theory, the overall aim of this study is to investigate how sporting organisations perceive sport for older adults. This includes the sporting organisation's current engagement with older adults and how sport professionals perceive their organisational readiness and ability to attract and/or retain older adults.

## Methods

In this descriptive study, we investigated the views of professionals working at NSOs and SSOs about sport participation for older adults. Six areas of sport for older adults were explored in this study. Areas 1–5 were informed by organisational change theory, whilst the sixth area was guided by previous research. Area one investigated how sporting organisations prioritised engaging older adults, compared to other population groups. This is important in order to investigate organisations' potential readiness to engage older adults. Areas two and three provide context for NSOs' and SSOs' current older adult engagement, by asking if sports had specific strategies (area two) or programmes (area three) that targeted older adults. Areas four and five explored NSOs and SSOs' perceptions about the benefits of (area four), and barriers to (area five), older adult active sport participation. This was undertaken to better understand what levers sport management could use to enact organisational change, either through a planned or paradoxical change. The final area (six) assessed the organisations' perceptions of potential modifications to sport that could attract and/or retain older adults to participate. The potential modifications suggested were informed by a qualitative study by Jenkin et al. (2016) to provide practical advice for organisations wanting to engage more older adults into sport.

The research questions were analysed and compared across sports that typically have high, medium and low levels of older adult sport participation. There has been no precedent to categorising data to study the perspectives that sporting organisations have about sport and older adults, as this topic remains unexplored. The survey instrument was specifically developed for this project. Comparing the responses from sports with differing levels of current participation has enabled a more explicit understanding of the organisational mechanisms that influence older adults' participation in sport, including organisational readiness and capacity to change priorities. As noted, in our discussion we have placed this in context of the extent to which sporting organisations are ready and willing to consider age-friendly or even age-active strategic choices.

### Study Design

In this cross sectional study, we used an online survey for sporting organisations, regarding sport participation for older adults. There is no defined “older adults” category in sport, with previous research on older adults and sport, including community sport, categorising older adults from 52 to 94 years old (e.g., Dionigi, 2006; Heo et al., 2013). For this project it was decided to use 50 years old as the lower cut-off to define older adults in the community sport context (Eime et al., 2016a).

### Sample

**Figure 1:**
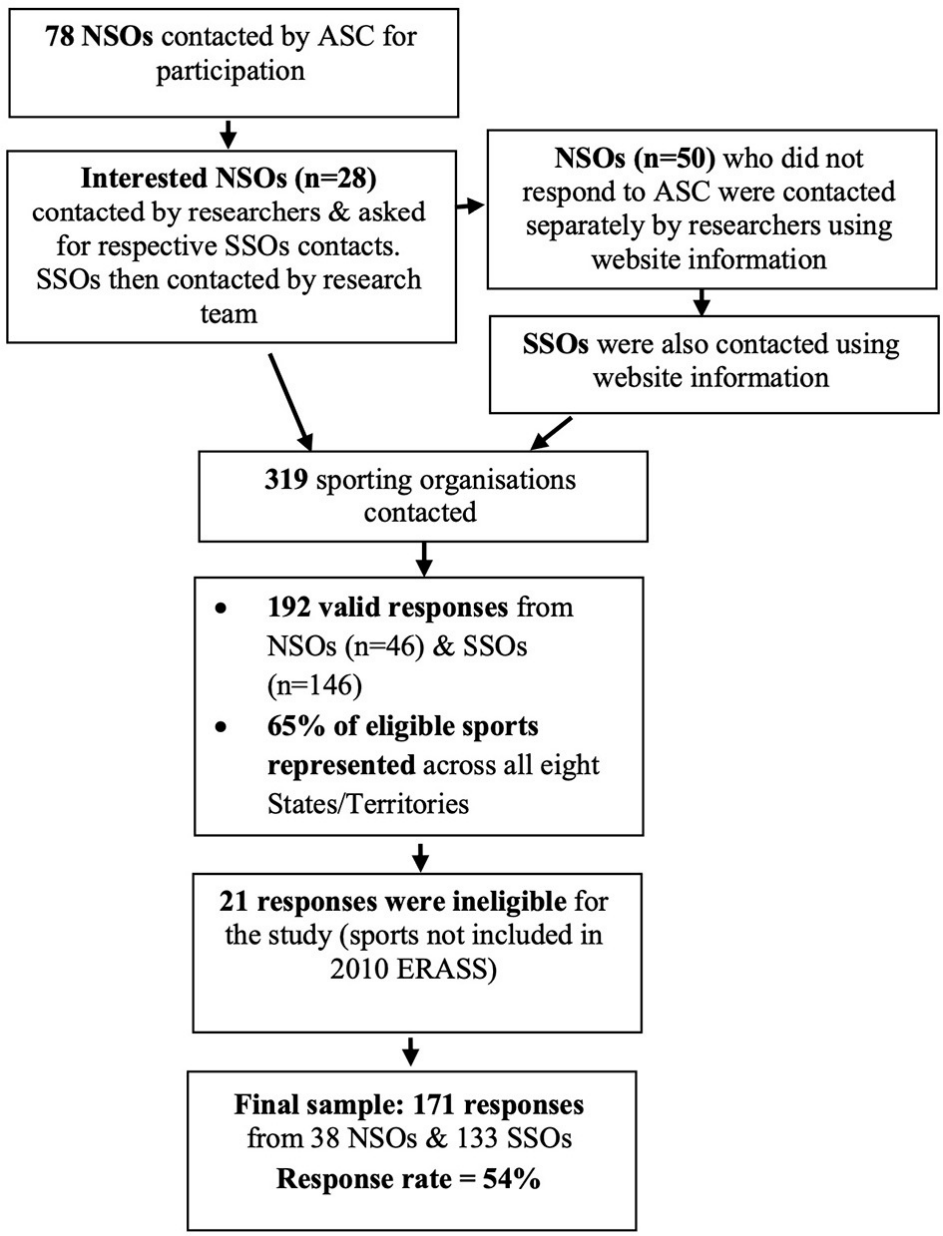
Flow chart detailing the recruitment process for the study.

The NSOs that are recognised by Sport Australia, and their affiliated SSOs, were included in this study. Of the total number of 93 NSOs, 78 NSOs and their affiliated SSOs were invited to complete the online survey. The remaining 15 NSOs were not invited to participate, as these were organisations that did not deliver community club-based specific sports, such as Australian University Sport and the Australian Paralympic Committee. The recruitment process is described in Figure 1.

The invited sporting organisations were recruited with support of SA. SA emailed relevant employees within each selected NSO to ask permission for the researchers to contact them.

There were 192 valid responses across 46 NSOs and 146 SSOs received. Responses were valid if all the questions were answered. There were 65% of eligible sports across all eight Australian States/Territories represented. To compare sports that have varying rates of older adult participation, sports were classified as having a low, medium or high older adult participation rate. This was determined using the sport participation data (participation in the previous 12 months) from the Australian Exercise, Recreation and Sport Survey (ERASS) data, an Australian Sport and the State/Territory Departments of Sport and Recreation initiative. Using the ERASS participation rates of people aged 50+ years who participated in Sport Australia recognised sporting organisations (Sport Australia, 2012), three classifications of sports were created: high (>0.5% of Australian older adult population), medium (< 0.5–>0.05% of Australian older adult population) or low rate (< 0.05% of Australian older adult population) of active sport participation amongst older adults. Twenty-one of the respondents were then classified as ineligible for this study, as they were from sports that were not included in the ERASS (Sport Australia, 2010), which was used to classify the three participation groups. As they could not be classified as high, medium or low participation sports, these responses were deemed incompatible for this study, as having no classification meant they would be unable to inform the learning process based on their existing level of older adult participation. Thus, 171 responses from 38 NSOs and 133 SSOs were analysed. Ethics approval for this study was obtained from the Victoria University Human Ethics Committee.

### Measures

Data were collected using an online survey hosted by the survey software platform Qualtrics. As previously discussed, the survey questions were informed by organisational change theory. For example, the *responses to the priorities'* question and the questions on *perceived benefits and barriers* could determine how much sporting organisations could see intrinsic motivations for change, rather than needing external pressure to do so. The survey item on *modifying the sport activity* can determine how agreeable and ready the sporting organisation was for potential change. These questions were also developed in collaboration with experts from SA, and then piloted with their employees for expert critical feedback, before being amended accordingly. The survey started with demographic questions related to the respondents' age, gender, job title, main role responsibilities, the type of organisation (National or State) and the length of time they had worked/volunteered in their respective sporting organisations.

This section was followed by six specific questions on *sport participation and older adults* (see complete questions in Tables 2–6). Firstly, respondents were asked to rate (on a five-point Likert scale ranging from very low to very high) their organisation's *level of priority across a range of population groups, including older adults*, as presented in Table 2. Respondents were then asked to reply “yes” or “no” to two items enquiring if their organisations had any *specific strategies or programmes for older adults*, as presented in Table 3. For this study, strategies were defined as “a long-term plan to attract older adults into your sport, and/or retain players as they age, which is not part of a specific older adults' programme.” A programme was defined as “a formal programme(s)/series of activities that are specifically designed for older adults.”

**Table 2:** Sporting organisations' level of priority on a three point Likert scale for different population groups.

**Survey item**		**High^d^ participation rate (%)**			**Medium^e^ participation rate (%)**			**Low^f^ participation rate (%)**			**Sample size**	**Statistical analysis**		
		**Low^a^**	**Neither^b^**	**High^c^**	**Low**	**Neither**	**High**	**Low**	**Neither**	**High**	**(*n*)**	**Kruskal- Wallis**	**Mann-Whitney U (*p* <0.017)**	**Effect size *r***
What is your sporting organisation's level of priority to increase sport participation for each of the following groups?	Primary school children (5–10 yrs)	7.5	15.1	**77.4**	9.1	3.0	**87.9**	11.1	22.2	**66.7**	170	^*^		
	Early to mid-teenagers (11–15 yrs)	5.7	11.3	**83.0**	4.0	8.0	**88.0**	0.0	5.6	**94.4**	171			
	Older teenagers (16–19 yrs)	7.5	24.5	**67.9**	5.1	18.2	**76.8**	0.0	5.6	**94.4**	170	^*^	Lo > Hi (participation rate)	0.36
	Adults (20–49 yrs)	7.5	30.2	**62.3**	10.0	30.0	**60.0**	0.0	11.1	**88.9**	171	^*^	Lo > Hi	0.31
	Older adults (50+ yrs)	13.2	**43.4**	**43.4**	21.2	37.4	**41.4**	**38.9**	**38.9**	22.2	170			
	Culturally and linguistically diverse communities (CALD)	20.8	**49.1**	30.2	13.1	31.3	**55.6**	11.1	**50.0**	38.9	170	^*^	Med > Hi	0.27
	Aboriginal and Torres Strait Islanders (ATSI)	20.8	**43.4**	35.8	17.0	39.0	**44.0**	5.6	38.9	**55.6**	171			
	People with a disability	13.5	42.3	**44.2**	11.0	26.0	**63.0**	**38.9**	**38.9**	22.2	170	^*^	Med > Hi Med > Low	0.21
	Women	0.0	28.3	**71.7**	3.0	16.0	**81.0**	0.0	22.2	**77.8**	171			
	Men	3.8	**49.1**	47.2	5.0	32.0	**63.0**	0.0	38.9	**61.1**	171			

a *Low = low or very low priority*.

b *Neither = neither high nor low priority*.

c *High = high or very high priority*.

d *High participation rate = >0.5%*.

e *Medium participation rate = >0.05– <0.5%*.

f *Low participation rate = < 0.05%.*

*These rates are the number of older adults actively participating in sport and were devised using the Exercise Recreation and Sport Survey (ERASS) 2010 dataset*.

*Percentages highlighted in bold represent the most agreed variable answer for that participation category*.

* *represents statistical significance (p < 0.05). Only significant Mann-Whitney U paired comparisons are listed. Due to there being three pairs of response rates to compare, the statistical significance for the Mann-Whtiney U test was set at p < 0.017*.

**Table 3:** Specific strategies and/or specific programmes for older adults.

**Survey item**	**High participation rate^a^ (%)**		**Medium participation rate^b^(%)**		**Low participation rate^c^ (%)**		**Sample size**	**Statistical analysis**		
							**(*n*)**	**Fisher exact (f)/ Chi square**	***p*-value**	**Cramer's *V* effect size**
Does your organisation currently have any specific sport participation strategies, which are not a component of a specific programme, for older adults?	Yes	No	Yes	No	Yes	No				
	30.2	**69.8**	28.0	**72.0**	16.7	**83.3**	171	1.27	0.57	0.09
Does your organisation currently have any specific sport participation programmes for older adults?	Yes	No	Yes	No	Yes	No				
	41.5	**58.5**	45.0	**55.0**	**50.0**	**50.0**	171	0.42	0.79	0.05

a *High participation rate = >0.5%*.

b *Medium participation rate = >0.05– <0.5%*.

c *Low participation rate = <0.05%*.

*These rates are the number of older adults actively participating in sport and were devised using the Exercise Recreation and Sport Survey (ERASS) 2010 dataset*.

*Percentages highlighted in bold represent the most agreed variable answer for that participation category. Statistical significance was set at minimum p < 0.05*.

**Table 4:** Potential organisational barriers on a three point Likert scale for older adults' sport participation.

**Survey item**		**High^d^ participation rate (%)**			**Medium^e^ participation rate (%)**			**Low^f^ participation rate (%)**			**Sample size**	**Statistical analysis**		
		**Disagree^a^**	**Neither^b^**	**Agree^c^**	**Disagree**	**Neither**	**Agree**	**Disagree**	**Neither**	**Agree**	**(*n*)**	**Kruskal- Wallis**	**Mann-Whitney U (*p* <0.017)**	**Effect size *r***
To what extent do you agree that the following issues could be barriers to increase participation in older people for your sport	**Barriers with agreement across all three participation groups**													
	Appropriate programmes for older players	19.2	17.3	**63.5**	25.0	14.0	**61.0**	16.7	11.1	**72.2**	170			
	Sufficient resources to manage programmes for this specific group	15.1	22.6	**62.3**	8.0	13.0	**79.0**	16.7	16.7	**66.7**	171			
	Sufficient resources to develop programmes for this specific group	17.0	20.8	**62.3**	8.1	15.2	**76.8**	11.1	22.2	**66.7**	170			
	Main focus is on other target groups	17.0	26.4	**56.6**	10.0	17.0	**73.0**	16.7	5.6	**77.8**	171	^*^	Med > Hi (participation rate)	0.20
	Specific competitions for older players	37.7	18.9	**43.4**	31.0	14.0	**55.0**	22.2	5.6	**72.2**	171			
	**Barriers with agreement across two participation groups**													
	Designated staff to manage programmes for this specific group	17.0	24.5	**58.5**	18.0	21.0	**61.0**	11.1	**50.0**	38.9	171			
	Designated staff to develop programmes for this specific group	20.8	24.5	**54.7**	19.0	24.0	**57.0**	11.1	**50.0**	38.9	171			
	Lack of demand from older adults to justify specific programmes for this group	**41.5**	30.2	28.3	33.0	23.0	**44.0**	27.8	22.2	**50.0**	171			
	**Barriers with disagreement/neither agree nor disagree across all three participation groups**													
	Suitable equipment for older players	**58.5**	22.6	18.9	**55.0**	27.0	18.0	**38.9**	27.8	33.3	171			
	Suitable facilities for older players	**60.4**	18.9	20.8	**54.0**	19.0	27.0	**44.4**	27.8	27.8	171			
	Concerns/difficulties about insuring older players	**69.8**	20.8	9.4	**56.0**	31.0	13.0	**55.6**	27.8	16.7	171			
	Main focus is on other age groups	39.6	**60.4**	0.0	26.0	**74.0**	0.0	22.2	**77.8**	0.0	171			

a *Disagree = strongly disagree/disagree*.

b *Neither = neither agree or disagree*.

c *Agree = agree/strongly agree*.

d *High participation rate = >0.5%*.

e *Medium participation rate = >0.05– <0.5%*.

f *Low participation rate = <0.05%*.

*These rates are the number of older adults actively participating in sport and were devised using the Exercise Recreation and Sport Survey (ERASS) 2010 dataset*.

*Percentages highlighted in bold represent the most agreed variable answer for that participation category*.

* *represents statistical significance of at least p < 0.05. Only significant Mann-Whitney U paired comparisons are listed. Due to there being three pairs of response rates to compare, the statistical significance for the Mann-Whtiney U test was set at p <0.017*.

**Table 5:** Potential organisational benefits on a three point Likert scale for older adults' sport participation.

**Survey item**		**High^d^ participation rate (%)**			**Medium^e^ participation rate (%)**			**Low^f^ participation rate (%)**			**Sample size**	**Statistical analysis**
		**Disagree^a^**	**Neither^b^**	**Agree^c^**	**Disagree**	**Neither**	**Agree**	**Disagree**	**Neither**	**Agree**	**(*n*)**	**Kruskal-Wallis**
To what extent do you agree that the following outcomes of increasing participation for older adults could be beneficial for your sport	Increase overall participation numbers	1.9	5.7	**92.5**	3.0	6.0	**91.0**	0.0	5.6	**94.4**	171	
	Engage with your older fan base	7.5	13.2	**79.2**	6.0	14.0	**80.0**	0.0	16.7	**83.3**	171	
	Be socially responsible and as such accommodate a growing older demographic in our society	3.8	22.6	**73.6**	2.0	19.0	**79.0**	0.0	27.8	**72.2**	171	
	Increase your older fan base	7.5	24.5	**67.9**	6.0	19.0	**75.0**	5.6	16.7	**77.8**	171	
	Develop positive role models for your younger players	11.3	34.0	**54.7**	9.0	26.0	**65.0**	0.0	22.2	**77.8**	171	

a *Disagree = strongly disagree/disagree*.

b *Neither = neither agree or disagree*.

c *Agree = agree/strongly agree*.

d *High participation rate = >0.5%*.

e *Medium participation rate = >0.05– <0.5%*.

f *Low participation rate = <0.05%*.

*These rates are the number of older adults actively participating in sport and were devised using the Exercise Recreation and Sport Survey (ERASS) 2010 dataset*.

Percentages highlighted in bold represent the most agreed variable answer for that participation category. *represents statistical significance of at least p < 0.05. Only significant Mann-Whitney U paired comparisons are listed. Due to there being three pairs of response rates to compare, the statistical significance for the Mann-Whtiney U test was set at p <0.017. *The meaning is described in italics underneath the table.

**Table 6:** Potential organisational modifications on a six point Likert scale to retain/attract older adults to sport.

**Survey item**		**High^d^ participation rate (%)**			**Medium^e^ participation rate (%)**			**Low^f^ participation rate (%)**			**Sample size**	**Statistical analysis**		
		**Disagree^a^**	**Neither^b^**	**Agree^c^**	**Disagree**	**Neither**	**Agree**	**Disagree**	**Neither**	**Agree**	**(*n*)**	**Kruskal-Wallis**	**Mann-Whitney U (*p* <0.017)**	**Effect size *r***
To what extent do you agree that the following potential modifications could help to retain current older players and/or attract new older players in your sport	**Barriers with agreement across all three participation groups**													
	Change the way your sport is advertised to older adults	2.0	8.2	**89.8**	4.2	18.8	**77.1**	5.9	23.5	**70.6**	162			
	Collaborate with community organisations	2.1	17.0	**80.9**	1.1	16.8	**82.1**	5.9	17.6	**76.5**	159			
	Collaborate with ageing/senior programmes	0.0	16.7	**83.3**	2.1	16.0	**81.9**	5.9	29.4	**64.7**	159			
	Increase flexibility of membership options	8.3	12.5	**79.2**	12.9	16.1	**71.0**	5.9	29.4	**64.7**	158			
	Introduce social play rather than competition	4.3	19.6	**76.1**	4.5	20.2	**75.3**	5.9	23.5	**70.6**	152			
	Introduce age specific social play categories (i.e., over 50 years)	6.5	13.0	**80.4**	6.6	19.8	**73.6**	12.5	25.0	**62.5**	153			
	Introduce age specific competition categories (i.e., over 50 years)	8.5	17.0	**74.5**	5.6	21.3	**73.0**	25.0	18.8	**56.3**	152			
	Shorter playing time	19.0	19.0	**61.9**	22.4	15.3	**62.4**	14.3	14.3	**71.4**	141			
	Introduce gender specific strategies or programmes	17.4	34.8	**47.8**	19.6	32.6	**47.8**	11.8	23.5	**64.7**	155			
	Lower the cost of participating	20.4	16.3	**63.3**	20.8	15.6	**63.5**	29.4	**35.3**	**35.3**	162	^*^	Med > Hi (participation rate)	0.22
	Shorter training sessions	12.5	42.5	**45.0**	12.0	25.3	**62.7**	0.0	42.9	**57.1**	137			
	**Barriers with agreement across two participation groups**													
	Decrease in level of physical contact	36.1	**44.4**	19.4	34.2	21.9	**43.8**	0.0	30.8	**69.2**	122			
	Lower frequency of training sessions	27.8	**47.2**	25.0	15.7	32.5	**51.4**	14.3	**42.9**	**42.9**	133			
	Lower frequency of matches	37.5	**40.0**	22.5	20.7	31.7	**47.6**	14.3	35.7	**50.0**	136			
	More accessible locations (i.e., near retirement villages)	15.9	34.1	**50.0**	19.3	34.1	**46.6**	**50.0**	31.3	18.8	148	^*^	Hi > Lo Med > Hi	0.33, 0.24
	Smaller playing size (i.e., court/pitch/oval)	25.8	**45.2**	29.0	35.6	21.9	**42.5**	23.1	23.1	**53.8**	117			
	Changes in equipment	27.5	**40.0**	32.5	**47.1**	28.7	24.1	**58.8**	35.3	5.9	144	^*^	Hi > Lo	0.36
	Increase in team size	36.4	**63.6**	0.0	**45.3**	30.7	24.0	30.8	**53.8**	15.4	121			
	Decrease in team size	33.3	**60.6**	6.1	**42.7**	32.0	25.3	30.8	**53.8**	15.4	121			
	Introduce a stronger focus on specific strength and conditioning programmes	18.2	**43.2**	38.6	15.6	**43.3**	41.1	17.6	**41.2**	**41.2**	151			
	**Barriers with no agreement across all three participation groups**													
	Improve accessibility (i.e., introduce ramps/ handrails)	19.5	**43.9**	36.6	35.6	27.6	**36.8**	41.2	**47.1**	11.8	145	^*^	Hi > Lo	0.36

a *Disagree = strongly disagree/disagree;*

b *Neither = neither agree or disagree;*

c *Agree = agree/strongly agree;*

d *High participation rate = >0.5%;*

e *Medium participation rate = >0.05– <0.5%;*

f *Low participation rate = <0.05%; These rates are the number of older adults actively participating in sport and were devised using the Exercise Recreation and Sport Survey (ERASS) 2010 dataset; Percentages highlighted in bold represent the most agreed variable answer for that participation category*.

* *represents statistical significance of at least p < 0.05. Only significant Mann-Whitney U paired comparisons are listed. Due to there being three pairs of response rates to compare, the statistical significance for the Mann-Whtiney U test was set at p < 0.017*.

The next survey section asked for respondents' level of agreement across a five-point Likert scale (strongly disagree to strongly agree) on *potential organisational barriers for older adults* participating in their sport (Table 4). This was followed by a section that asked respondents for their level of agreement of *potential benefits their organisations could derive from engaging older adults* across a five-point Likert scale (strongly disagree to strongly agree), detailed in Table 5. The final section asked respondents to indicate their level of agreement on a five-point Likert scale (from strongly disagree to strongly agree plus a “not applicable” category) with potential *modifications to their sport that could attract and/or retain older adults* (Table 6).

### Data Analysis

Data were analysed using the Statistical Package for the Social Sciences (SPSS). Percentages across response categories for the demographic survey items are reported. Due to some low cell counts amongst Likert responses, five-point response scales were collapsed into three-point response scales. Responses were compared for sports with low, medium and high older adult participation rates, using Chi-square tests across all non-demographic survey items. For ordinal data, Kruskal-Wallis tests were used to compare response distributions across the full five response options between the three participation categories of older adult participation. Due to the sample size and distributions, the Monte Carlo method was used to calculate accurate significance values. For significant Kruskal-Wallis results, *post-hoc* Mann-Whitney U tests were used to compare each of the three participation category pairs. To reduce the risk of type 1 error for the three Mann-Whitney U tests, significance was set at *p* < 0.017. For significant results, an effect size was calculated using Pearson *r*. For the nominal data, Chi square tests, and Fisher's exact test when more than 20% of expected cell counts were below five or any cells were below one, were used to compare frequencies between the three participation categories. Significance was set at < 0.05 for the nominal data, and effect size was calculated using Cramer's *V*.

## Results

The majority of the respondents were male (64%), with a mean age of 44 years (SD = 12.92) and worked in a variety of roles, including Chief Executive Officers, Participation Managers and Association Secretaries. The majority of respondents (76%) had a focus on both strategic development and programme delivery, whilst 12% were responsible for strategic delivery only. Additionally, 6% of respondents were responsible for programme delivery only and 6% had a focus on other responsibilities. Forty-five per cent of respondents had worked in their respective organisations for 1–5 years. There was a similar mean age across the three groups, with substantially more male respondents in the low participation group compared to the other two groups.

Tables 2–6 compare the survey item responses across the three groups of sporting organisations, for each of the six main survey sections. Tables 2–6 also display sample size for each item, plus the relevant statistics for each type of data.

### Sporting Organisations' Prioritising of Older Adult Participation

Older adults were not considered a high priority population group when aiming to increase sport participation (see Table 2), with less than half of the sports reporting older adults a priority for them.

Most sports, including those with high and medium older adult participation, were most likely to prioritise participation specifically for children, youth and women. Thus, regardless of the number of older adults participating in each sport, the prioritising of children and youth was consistent across all sporting organisations. There was no sense of urgency to engage in creating age-friendly sporting programmes and environments, let alone specifically focus on older adults as a priority demographic group.

Between group priority differences for three population groups were statistically significant: primary school children, CALD and people with a disability. Low older adult participation sports significantly prioritised both older teenagers and adults more than high older adult participation sports.

### Older Adult Focused Sport Strategies and Programmes

Whilst most sporting organisations did not have specific strategies for older adult active participation (see Table 3), more high and medium participation sports had specific strategies than low participation sports. Considerably more sporting organisations had specific participation programmes for older adults than strategies. There was no evidence that these programmes had been developed in the broader context of physiological ageing.

### Sporting Organisations' Perceptions of Barriers to Engaging Older Adults

There was widespread agreement across the three participation groups on the main organisational barriers for participation (Table 4). The most prominent barriers were lack of appropriate programmes for older players, and insufficient resources to manage and develop older adult programmes. There was also consensus across the participation groups regarding barriers they disagreed with. For example, most respondents disagreed that suitable equipment or facilities for older adults were a barrier to participation. It seems that there is a distinct need for (and opportunity to) better educate sport managers about the ways in which older adults can be better understood and communicated to in regard to their physical, cognitive and sensory needs and wants.

### Sporting Organisations' Perceptions of Benefits to Engaging Older Adults

The majority of all sporting organisations in this study agreed with all of the provided organisational benefits of engaging older adult**s** in sport (see Table 5). Particularly prominent was the opportunity to increase their overall participation numbers, whilst over three-quarters of all respondents agreed that organisations could engage with their older fan base. In context of the strategic options model, most sporting organisations are missing the market opportunity to reap these potential benefits.

### Sporting Organisations' Perceptions of Potential Modifications to Sport to Attract and/or Retain Older Adults

Twenty-one potential sporting modifications to attract and/or retain older adults into sport were suggested to respondents (Table 6). The most popular modifications across the three participation categories were changing the way their sport is advertised and collaboration with community/ageing organisations. Introducing age specific social play opportunities was also a popular modification for high participation sports. For medium participation sports, introducing social play rather than competition was widely agreed upon, whilst providing a shorter playing time was a popular modification for low participation sports.

There were statistically significant differences between the older adult participation groups for four potential modifications. Significantly more high than low participation sports reported that more accessible locations, improving accessibility and changes in equipment, could be possible modifications for their respective sports.

## Discussion

Through a lens of sport marketing, this study investigated the perceptions of Australian NSOs and SSOs regarding six different areas that related to older adult sport participation: (1) what is the level of priority of different population groups for sporting organisations; (2 and 3) if sporting organisations have current sport specific participation strategies or programmes for older adults; (4 and 5) what are organisational benefits of, and barriers to, older adult sport participation; and (6) what are potential modifications that could attract and/or retain older adults in their respective sports. Potential differences between sports that had high, medium or low levels of older adult participation were examined. Overall, there was broad agreement across most of the variables, but there were some differences, which will be further discussed below. We use terminology that was presented in Table 1 to further contextualise our results. To that end we will describe sporting organisations and/or their strategies towards engaging with older adults as passive, age-friendly, or age-active.

For all sports, regardless of their current rate of older adult participation, older adults were not considered a high priority group compared to children, youth and women. Similarly to previous research, children were considered the highest priority in regard to recruitment into sport (Eime et al., 2016b; Österlind, 2016). In light of the significant proportional population growth of the older adult segment in most Global North, this can be described as a myopic attitude towards sport marketing. The market is there for the taking, but the strategic choice has largely been one of passive or reactive nature, such as the Better Ageing Grants introduced by Sport Australia in 2018.

However, the development of modified sport products for older adults, which could be classified as developing age-friendly environments or even age-active sport products, implies that some sporting organisations at least consider catering for this population group, but delivery of modified sport appears to be *ad hoc* and often heavily dependent on external funding. A paradoxical organisational change (Slack and Hinings, 1992) in sport policy to mandate that sporting organisations should take a more strategic and evidence informed approach to catering for older adults, may enable greater sustainability and availability of these programmes. This may then subsequently result in increased participation.

It was identified that few sporting organisations had specific strategies to increase older adult participation, with more organisations having specific programmes for this age group. Slightly more low participation sports than high participation sports have specific programmes for older adults, which may mean that these sports may not be suitable for older adults in their traditional format. Therefore, specific modifications may be required to enable and encourage older adult participation. Similarly, it is to be expected that more high and medium participation sports have specific strategies for older adults than low participation sports, as formal strategies usually underpin an organisation's priorities, which are largely influenced by external sport policy (Eime et al., 2016a). Providing specific programmes for different population groups, regardless of their priority level, is often easier than embedding these population groups into formal strategies. This may explain why fewer organisations had specific older adult participation strategies than programmes. However, for older adults to become a higher priority group for sporting organisations, specific (age-friendly or age-active) strategies and engagement policies (Westerbeek and Eime, 2021) that operationalise this priority, are required (Stroud and Walker, 2013).

There are examples of strategies and policies aimed at increasing participation for other specific underrepresented demographic groups, which organisations could use as an example for increasing older adult participation. For example, the specific sport strategies and policies driving increased participation in sport for women and girls, in both leadership and playing, in Australia and the UK (UK Sport and Sport England, 2016; VicSport, 2017), are impacting change with participation for women and girls increasing at greater rates than for boys and men, particularly in male-dominated sports (Casey et al., 2019; Eime et al., under review^1^). This is as a result of paradoxical organisational change in sport (Slack and Hinings, 1992; Skinner et al., 1999; Amis et al., 2004). If similar strategies were developed and implemented for older adults, including the creation of more age-friendly sporting environments, and appropriate age-active programmes, then we also may see considerable change and increased visibility of older adults in community sport, both as players and as volunteers.

There was widespread agreement amongst the sports on the organisational barriers of engaging older adults in sport. Low and medium participation sports reported a (statistically significant) focus on younger target groups, rather than high participation sports. It can be argued, in that regard, that an age-active (or one could argue, active ageing) marketing focus most likely would lead to achieving higher prominence in that market. Lack of appropriate older adult programmes was a key barrier in this study, and has been previously identified as a barrier for both older adults (Jenkin et al., 2016) and another underrepresented group, disabled children (Shields et al., 2011). Another barrier was insufficient resources to accommodate older adults. As Robertson et al. (2018) noted, sporting organisations cannot cater for all population groups, due to limited resources. Thus, unless there is a paradoxical organisational change (Slack and Hinings, 1992) to either mandate focus (through policy or incentives) on older adults, or resourcing for developmental support, these organisational barriers are unlikely to be mitigated.

All sporting organisations agreed that availability of suitable equipment or facilities was not an obstacle. However, suitable facilities has been previously identified by older adults themselves, as obstacles to their participation (Jenkin et al., 2018). This suggests there may be a potential discord between the organisational and older adult perspective on barriers to their participation. This finding shows the importance of engaging with the target market directly, rather than basing organisational views on other data sources.

Despite the lack of appropriate programmes and insufficient resources to cater for older adults, most sporting organisations recognised the opportunity to increase overall participation numbers. As all sporting organisations with a grassroots participation focus are targeting increased participation (Eime et al., 2015b), it would seem logical for sporting organisations to diversify their membership and extend their market reach to achieve this increase. It seems in that regard, the older adult market segment is not (yet) attractive enough for sporting organisations, to organically bring them into their marketing scope of attention. Further research into reasons why sporting organisational professional staff hold that view is required.

The two modifications that received widespread agreement across the sports were changes to the way sports are marketed and advertised and how to better collaborate with other relevant organisations. Bespoke and targeted marketing has been highlighted as a successful means towards recruiting children into modified sport (Eime et al., 2015a). However, the lack of diversity in sport target marketing has been identified by research into older adults (Jenkin et al., 2021), in addition to other underrepresented population groups, such as those from CALD communities (Sawrikar and Muir, 2010). The recognition of a lack of targeted marketing as a barrier to older adult participation, also reflects that older adults are not a priority segment.

Collaborations with community and/or ageing organisations was also considered as an important option for better focus. Some collaborations between community and sporting organisations that promote participation for older adults, for example, the Walking Basketball programme between Basketball Victoria and VicHealth, have provided appropriate participation opportunities and demonstrated successful partnerships to engage underrepresented groups (Basketball Victoria, 2017). These organisations have demonstrated a readiness to change (Oakland and Tanner, 2007) and/or their employees have been prepared to enact this change to actively engage older adults in their respective sports. More long-term collaborations of this kind would extend the range of opportunities for older adults to participate in sport. Such collaborations could also help to infuse the requisite knowledge about creating age-friendly environments into sporting organisations, and mitigate the lack of resources barrier that was identified in this study.

It is argued that the results from this study can provide overarching recommendations, such as developing bespoke age-active marketing campaigns, products and services, in addition to developing organic community collaborations. The majority of sporting organisations reported that older adults were not a high priority population group and most of the organisations in this study faced similar barriers, identified similar benefits and believed similar modifications could attract and/or retain older adults across different types of sports. The authors recognise that sport is not for everyone and that some age specific modifications, such as reduced physical contact, may need to be sport specific to increase overall older adult sport participation. However, some similar overarching strategies that can make sport across the board more attractive and accessible for older adults, can result in more of this population group having the opportunity to be physically active through sport. Conversely, as the organisational priority level to target older adults is currently low, it is unlikely that older adult sport participation will increase in the short term, unless there is a significant organisational change in the participation priorities set by the leaders of sporting organisations. This is unlikely to happen unless a paradoxical change (Slack and Hinings, 1992) is initiated at the national sport policy level. Despite there being other aspects within a sporting organisation that could enable opportunities for older adults, we propose that mandated policy will be the biggest organisational change lever. Policy usually directs organisational focus, development or recruitment of (bespoke marketing) knowledge, funding and priorities, which permeates all levels of an organisation and its executive management key performance indicators. As respondents reported lack of resources and low priority for older adults as barriers, it can be concluded that the majority of sporting organisations are not (yet) in a readiness to change state (Oakland and Tanner, 2007). Planned change to increase older adult sport participation in the near future is unlikely. It can therefore be argued that national sport policy will instigate focus on older adults as a priority target market and as such, initiate paradoxical change in sporting organisations.

### Strengths and Limitations

This study sought the perspectives of representatives from NSOs and SSOs across Australia on sport participation for older adults. Although the response rate was high and a wide variety of sports and States/Territories were represented, there may be some self-selection response bias, that is, those organisations with a stronger interest in older adults may have been more likely to respond. Therefore, the current study cannot be assumed to be reflective of the opinions of all NSOs and SSOs in Australia, and the study may provide a foundation for international comparison rather than generalisability. A further limitation of the study, which is inherent in quantitative research, was that the data was limited to the response options offered. Future research would benefit from the inductive, contextual and nuance rich data that comes from qualitative inquiry, to gain a richer insight into the “why” and “how” questions of sport policy development for older adults.

Global populations are ageing rapidly and ageing is often associated with a decline in health, which is why older adults are an important population group for sporting organisations to target to increase their participation rates. However, it seems that sporting organisations seem less concerned about achieving health outcomes (which is not one of their primary objectives) and more on increasing their participation rates (which is). Therefore, sporting organisations currently prioritise younger age groups, resulting in few appropriate participation opportunities for older adults. However, with an ageing population and the growing importance of the sport for health concept, there is a great opportunity for sporting organisations to take advantage of the growing older adult market. This research is the first to gain input from a wide representation of sporting organisations on older adult sport participation. It also provides marketing and product development insights for further developing age-friendly sporting environments and age-active modified sport products.

### Conclusion

Few sporting organisations and their management have strategically focused attention to the growing market of older adults. In line with organisational change theory, a paradoxical sport policy change is required to increase the focus on bringing older adults into sports clubs as active participants. This policy change should be underpinned by an understanding that to truly enable bringing more older adults into sport, a physiological ageing perspective needs to drive strategic decision making. This in turn will help a move towards providing age-friendly environments and age-active products and services. Legislated policy and resource support, including funding opportunities, would help sports and their management to specifically focus their capacity on older adults, to develop and deliver appropriate opportunities for their active participation. Most sporting organisations recognised the benefits of engaging with older adults, but to date it seems that benefits do not yet outweigh the costs of changing or expanding focus to multiple target markets. Young people in that regard, still seem to be an easier and more attractive target market for sport. However, increasing older adults' active participation in sport, through playing and volunteering, will not only benefit the sport sector, but also the health of individuals and communities. To achieve such benefits, government and sport need to work hand in hand to provide these opportunities.

## Data Availability Statement

The datasets presented in this article are not readily available because data to be kept confidential. Requests to access the datasets should be directed to Claire Jenkin, c.jenkin@herts.ac.uk.

## Ethics Statement

The studies involving human participants were reviewed and approved by Human Research Ethics Committee, Victoria University. Online informed consent for participation was provided by respondents prior to completion of the survey, in accordance with the national legislation and the institutional requirements.

## Author Contributions

CJ co-designed the survey, recruited the respondents, analysed the results, and co-wrote the paper. JvU co-designed the survey and developed the writing of this paper. GO'S and JH supported the data analysis. RE supported the study design and developed the writing of this paper. HW supported the study design and co-wrote the paper. All authors contributed to the article and approved the submitted version.

## Funding

CJ was supported by a Sport Australia–Victoria University Ph.D scholarship. JvU was supported by a Sport Australia–Victoria University Senior Research Fellowship.

## Conflict of Interest

The authors declare that the research was conducted in the absence of any commercial or financial relationships that could be construed as a potential conflict of interest.

## Publisher's Note

All claims expressed in this article are solely those of the authors and do not necessarily represent those of their affiliated organizations, or those of the publisher, the editors and the reviewers. Any product that may be evaluated in this article, or claim that may be made by its manufacturer, is not guaranteed or endorsed by the publisher.

## Footnotes

^1^Eime, R., Harvey, J., Charity, M. and Westerbeek, H. (under review). Participation of women and girls in traditionally male-dominated sports. *Int. J. Sport Policy Politics*.
